# Concyclic CH-π arrays for single-axis rotations of a bowl in a tube

**DOI:** 10.1038/s41467-018-06270-6

**Published:** 2018-09-17

**Authors:** Taisuke Matsuno, Masahiro Fujita, Kengo Fukunaga, Sota Sato, Hiroyuki Isobe

**Affiliations:** 1 Department of Chemistry, The University of Tokyo, Hongo 7-3-1, Bunkyo-ku, Tokyo, 113-0033 Japan; 2 JST, ERATO, Isobe Degenerate π-Integration Project, Hongo 7-3-1, Bunkyo-ku, Tokyo, 113-0033 Japan; 3 Department of Chemistry, Tohoku University, Aoba-ku, Sendai, 980-8578 Japan

## Abstract

The hydrogen bond is undoubtedly one of the most important non-covalent interactions. Among the several types of the hydrogen bonds, the CH–π interaction is a relatively new notion that is being recognised in chemistry and biology. Although the CH–π hydrogen bond and conventional hydrogen bonds share common features such as directionality, this weak interaction has played a secondary role in molecular recognition. In this study, we have devised a host–guest complex that is assembled solely by the CH–π hydrogen bonds. Multivalent interactions of a bowl-shaped hydrocarbon with its peripheral hydrogen atoms are made possible via CH–π hydrogen bonds by adopting a tubular hydrocarbon as a host for their enthalpy-driven complexation. Concyclic arrays of weak hydrogen bonds further allow dynamic rotational motions of the guest in the host. Solid-state analysis with crystallographic and spectroscopic methods reveal a single-axis rotation of the bowl in the tube.

## Abstract (web summary)

The weak and directional CH-π hydrogen bond has rarely been exploited in the design of supramolecular complexes and molecular machinery. Here, the authors construct a bowl-in-tube complex stabilized solely by concyclic CH-π hydrogen bonds, and show that the guest exhibits single-axis rotational motion despite tight association with the host.

## Introduction

Among the various types of hydrogen bonds^1^, the CH–π interaction is uniquely characterised by its weak bonding governed largely by dispersion forces^2,3^. Although the CH–π hydrogen bond normally plays a secondary role in molecular recognition, reinforcement with other interactions such as hydrophobic effects has allowed this weak force to encapsulate non-polar hydrocarbon guests in capsule-like hosts without freezing their dynamic motions. Despite the tightened host–guest association in polar media, for instance, the CH–π hydrogen bonds permitted tumbling motions of the guests in the host^4^. Further control over the motions such as directionality remains an unexploited subject, which should benefit the future design of molecular machinery^5^. However, controlling motional directionality with weak non-covalent bonds is naturally challenging.

**Fig. 1:**
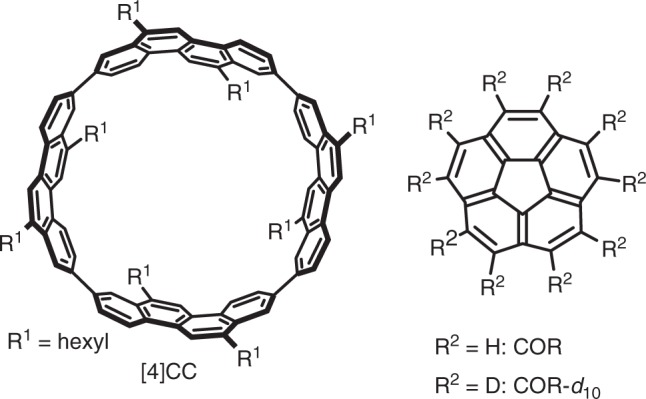
A tubular host ([4]CC) and a bowl-shaped guest (COR)

Here we report a bowl-in-tube complex of aromatic hydrocarbon molecules (Fig. 1)^6,7^. Solution phase studies with the aid of theoretical results reveal the fundamental association behaviours, and solid-state crystallography and spectroscopy reveal the unique dynamics. Solution phase analyses in non-polar media disclose a tight association of a 1:1 complex, which is driven by a large association enthalpy of –7 kcal mol^–1^. The theoretical studies show that the association enthalpy is gained by multiple CH–π hydrogen bonds arrayed in a concyclic arrangement. In the crystalline solid state, a 1:2 host–guest structure is unveiled to show concentric electron distributions of the disordered guests to indicate the presence of rotational freedom. Solid-state ^2^H NMR spectroscopy reveals the single-axis rotation of the entrapped hydrocarbon bowls. This study demonstrates that when embedded in elaborate molecular designs, the directional CH–π hydrogen bonds can assemble tight supramolecular complexes with motional dynamics.

## Results

### A 1:1 complex in solution

**Fig. 2:**
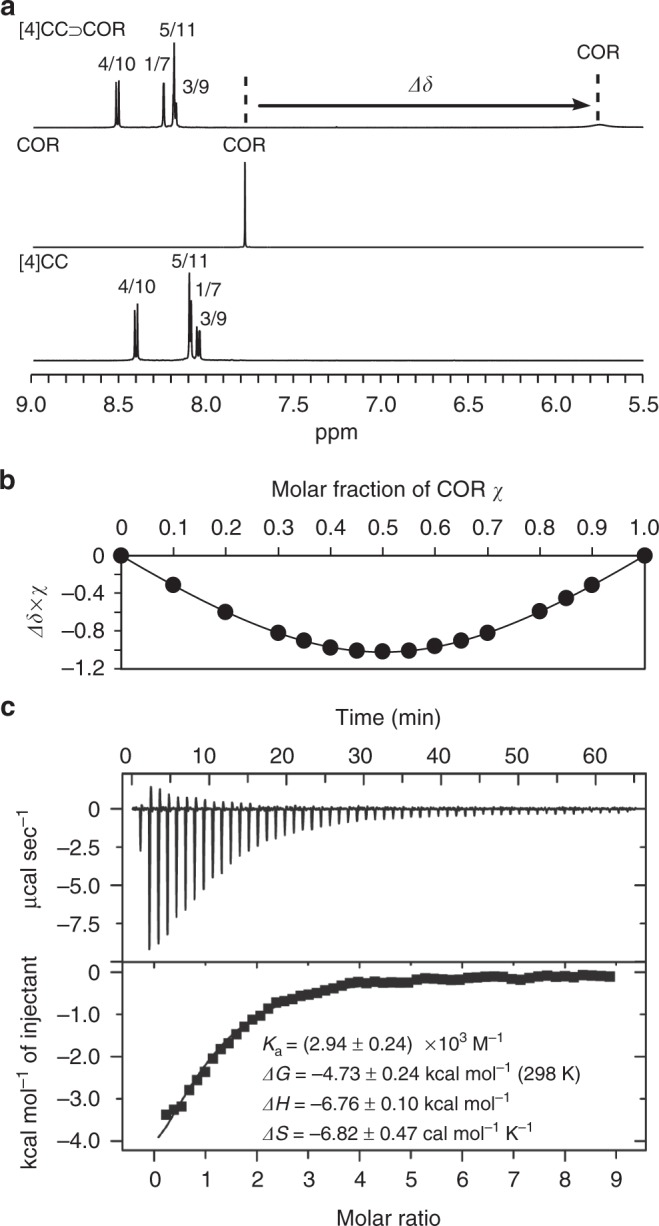
Complexation of [4]CC and COR in dichloromethane. **a** ^1^H NMR spectra at 298 K. See ref. ^8^ for the assignments of [4]CC. **b** Job plot analysis from ^1^H NMR spectra at total concentration of 1 mM to show the formation of 1:1 complex ([4]CC⊃COR). **c** Representative ITC data from quintuple titrations with thermodynamic parameters showing their average values and standard deviations

The hydrocarbon host in this study was (*P*)-(12,8)-[4]cyclo-2,8-chrysenylene (denoted as [4]CC)^6,8^, and the hydrocarbon guest was corannulene (denoted as COR) (Fig. 1)^9,10^. We first noticed their complexation from the resonance shifts in the ^1^H NMR spectra upon mixing these two molecules in dichloromethane-*d*_2_. As shown in Fig. 2a, a large up-field shift (*Δδ* = –2.04 ppm) was observed with the ^1^H resonance of COR, and downfield shifts of aromatic resonances of [4]CC were observed. The shielding/deshielding effects observed were consistent with the bowl-in-tube structure of [4]CC⊃COR that was revealed by theoretical and crystallographic analyses (see below). By performing a Job plot analysis with the most affected COR resonance, we thus determined 1:1 stoichiometry of the host–guest complex in solution (Fig. 2b). Broadening of the COR resonance was ascribed to a slow in-and-out exchange process with a relatively high energy barrier (*ΔG*^‡^) of + 11.0 kcal mol^–1^ (243 K; Methods)^11^. This high energy barrier was effective to broaden the COR resonance but could not separate the resonances of unbound and bound species at ambient temperature^12,13^.

Detailed thermodynamics of the bowl-in-tube complexation in dichloromethane was revealed by isothermal titration calorimetry (ITC) (Fig. 2c)^13,14^. Multiple titration experiments were performed in quintuple to afford the association constant of *K*_a_ = (2.94 ± 0.24) × 10^3^ M^–1^, and enthalpy-driven complexation of [4]CC⊃COR was revealed with the thermodynamics parameters of *ΔG* = –4.73 ± 0.24 kcal mol^–1^, *ΔH* = –6.76 ± 0.10 kcal mol^–1^ and –*TΔS* = +2.03 ± 0.14 kcal mol^–1^ (298 K). The COR guest possesses 10 CH bonds that are radially projected outward from the bowl centre. Thus, assuming 10 CH–π contacts for the complexation, we can estimate an enthalpy gain of –0.7 kcal mol^–1^ for one CH–π contact, which is reasonably expected for the weak CH–π hydrogen bonds^2^. We performed an identical analysis with deuterated COR (COR-*d*_10_; 80%D)^15^ to find negligible isotope effects for the complexation (*K*_a_ = (3.17 ± 0.09) × 10^3^ M^–1^, *ΔG* = –4.77 ± 0.21 kcal mol^–1^, *ΔH* = –6.61 ± 0.10 kcal mol^–1^ and –*TΔS* = +1.84 ± 0.10 kcal mol^–1^; 298 K) (Supplementary Fig. 4). The result was also consistent with a previous report on CH–π hydrogen bonds being insensitive to equilibrium isotope effects^16^.

### Theoretical studies of CH–π hydrogen bonds

**Fig. 3:**
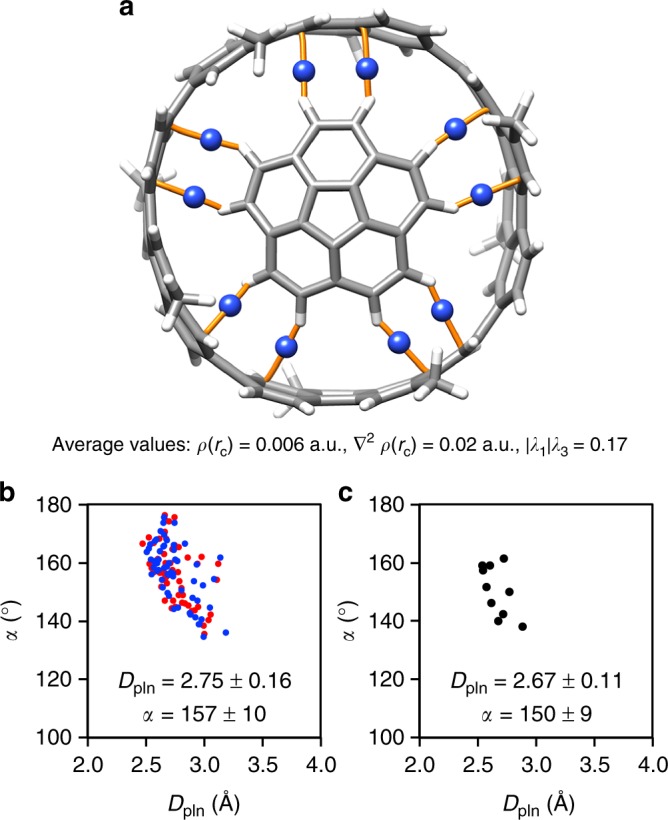
The CH–π hydrogen bonds in the bowl-in-tube complex. **a** Topological analyses of the electron density of Me-[4]CC⊃COR with the AIM method locating the 10 intermolecular bond paths (orange) with (3,–1) BCPs (blue), confirming the presence of the CH–π hydrogen bonds. The geometry was obtained by the DFT calculations [LC-BLYP/6-311 G(d)]. **b** A scatter plot of *D*_pln_ and *α* of COR_cent_ from crystal data of [4]CC⊃(COR)_2_ (red) and [4]CC⊃(COR-*d*_10_)_2_ (blue). For reference plots of typical CH–π hydrogen bonds, see Fig. 7 in ref. ^29^. **c** A scatter plot of *D*_pln_ and *α* of the optimised geometry of Me-[4]CC⊃COR

Theoretical calculations provided insights into the structures and the chemical nature of the bowl-in-tube assembly. With a methyl-substituted model of Me-[4]CC⊃COR, we first screened the level of theory among representative DFT methods for the reproducibility of the association enthalpy. Taking account of previous DFT investigations of the [4]CC host for a fullerene guest^17,18^, we adopted a common basis set of 6-311 G(d) with nine methods with long-range corrections. Despite the wide range of association energies between +0.43 and –32.2 kcal mol^–1^ (Supplementary Fig. 5), all the methods converged to afford a unique bowl-in-tube structure (Fig. 3a). After counterpoise corrections of basis set superposition errors (BSSE) and polarisable continuum model (PCM) solvation with dichloromethane, an association energy (*ΔE*) of –9.17 kcal mol^–1^ was obtained from the LC-BLYP method, which reproduced the experimental association enthalpy (*ΔH* = –6.76 kcal mol^–1^). The observed matching (*ΔE* = *ΔH*) as well as the functional dependency of energy deviations was similarly observed with van der Waals complex of [4]CC and C_60_^17^, and we believe that the long-range exchange corrections implemented with the LC-BLYP method can be an appropriate method to elucidate the association behaviours of large curved π-systems^18^.

The atoms-in-molecule (AIM) analysis for the topological analyses of the electron density [*ρ*(*r*_c_)] on the bowl-in-tube structure revealed the presence of the CH–π hydrogen bonds^19^. As shown in Fig. 3a, ten intermolecular bond paths with (3, –1) bond critical points (BCP) were found between the COR hydrogen atoms and the [4]CC carbon atoms in the structure optimised by LC-BLYP/6-311 G(d). Representative measures of *ρ*(*r*_c_), ∇^2^*ρ*(*r*_c_) and |*λ*_1_|/*λ*_3_ were then derived at the BCP to show that they were electronically within a characteristic range expected for the hydrogen bonds [0.002 < *ρ*(*r*_c_) < 0.034, 0.02 < ∇^2^*ρ*(*r*_c_) < 0.14 and |*λ*_1_|/*λ*_3_ < 1]^1,20,21^. Structurally, the optimised CH–π geometries also fell within a reasonable range expected for the CH–π hydrogen bonds (see below). As expected for hydrogen bonded assembly, the electron density difference maps showed a decrease in density around the bridging proton with an increase on the counterpart acceptor π systems (Supplementary Fig. 6)^22^. Thus, the AIM analysis showed that the bowl-in-tube complex was assembled by the 10 CH–π hydrogen bonds that anchored the bowl periphery to the tube wall.

The theoretical bowl-in-tube structure shown in Fig. 3a was also proven appropriate as a time-average structure in solution. The ^1^H resonance of COR in [4]CC experimentally appeared as a singlet at 3.57 ppm (Methods section; Supplementary Fig. 2), and the gauge-independent atomic orbital (GIAO) calculations of the COR resonance^23^ predicted a chemical shift of 3.86 ppm for the bowl-in-tube structure (Supplementary Fig. 7). Contrarily, another possible structure having convex–concave contacts predicted the COR chemical shift at 5.99 ppm. Although the solution phase structure of [4]CC⊃COR is dynamically fluctuating via rotations and in-and-out exchanges, the stable bowl-in-tube structure can serve, spectroscopically, as the time-average structure of the COR molecule rotating in [4]CC.

### Bowl-in-tube complex in crystals

**Fig. 4:**
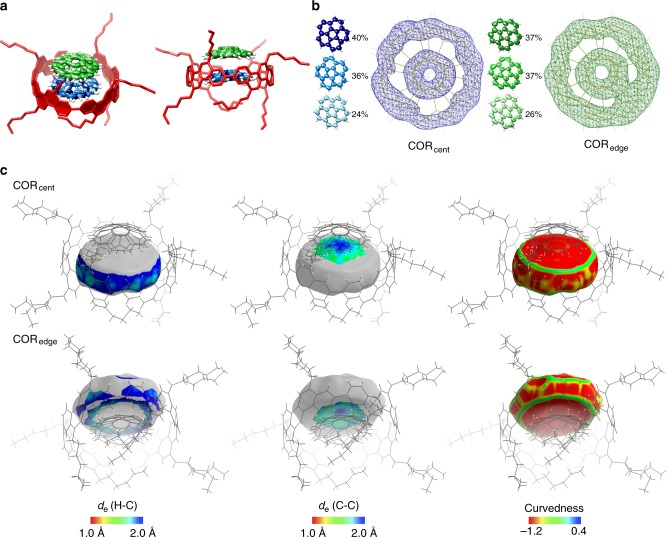
Crystal structures of [4]CC⊃(COR)_2_. **a** Molecular structures. One representative conformation of alkyl chains of the highest populations is shown for clarity. Thermal ellipsoids of COR are scaled to enclose 50% probability. Colour code: [4]CC = red, COR_cent_ = blue, COR_edge_ = green. **b** Contour electron density mappings (2*F*_*o*_–*F*_*c*_, RMSD 1.5*σ*), and disordered structures of COR. **c** The Hirshfeld surfaces of COR

Although we failed to obtain single crystals from a 1:1 mixture of [4]CC and COR, a single crystal was finally obtained in the presence of excess amount of the guest. Diffraction experiments were then performed with monochromated X-rays (SPring-8 BL38B1), and the crystallographic analysis revealed the molecular structures. Unexpectedly, we found a 1:2 host–guest complex of [4]CC⊃(COR)_2_ in the crystal (Fig. 4). We also obtained a single crystal with COR-*d*_10_, and negligible structural differences were noted (Supplementary Fig. 8). Among two entrapped COR guests, one was located at the central position of the tubular host (COR_cent_; blue molecules in Fig. 4a), and the other was located above it (COR_edge_; green molecules in Fig. 4a). Each COR guest was assigned as three disordered structures of different orientations (Fig. 4b). Contour maps for the raw electron densities of the disordered structures are also shown in Fig. 4b (2*F*_*o*_ − *F*_*c*_ maps at RMSD 1.5*σ*)^24^. The maps show concentric distributions of electrons on the COR atoms. The observation indicated the presence of single-axis rotations of the COR guests in the [4]CC host, which was confirmed by solid-state NMR analyses (see below).

Detailed intermolecular contacts in [4]CC⊃(COR)_2_ were revealed by the Hirshfeld surface analysis with *d*_e_ and curvedness mappings (Fig. 4c)^25,26^. The *d*_e_ mappings show the distance from the surface to the external atoms, and the curvedness mappings show the geometric inflection of the surface. The *d*_e_ mappings for H-C contacts of COR_cent_ visually demonstrated the presence of CH–π contacts between COR and [4]CC, whereas the CH–π contacts were not observed in the *d*_e_ mappings of COR_edge_. On the other hand, C-C contacts were present for both COR_cent_ and COR_edge_ molecules at their interface, indicating π-stack assembly of these two molecules. In the pristine single crystal, COR molecules were packed with CH–π and slipped π-stack contacts^27^. By occupying the CH periphery of COR, the tubular host thus reduced the chance of an edge-to-face CH–π contact and enhanced the π-stack motif with improved alignments. The COR_edge_ molecule might thus have filled the crystalline space with the concave–convex complementarity for the effective crystal growth. The curvedness mappings revealed smoothly curved surfaces at the interfaces of COR_cent_/[4]CC and COR_cent_/COR_edge_^26^, which should benefit the single-axis rotations of COR (see below). The concave-convex stack also highlights another uniqueness of the bowl-in-tube structure. Intrinsically, the unbound [4]CC host with *D*_4_ chirality does not have anisotropy along the tubular axis. However, by inserting the anisotropic bowl in the *D*_4_ tube, we create directionality in the tube axis. This symmetry breaking process is similar to the one that occurs upon insertion of a bolt into a nut and does not occur with an isotropic guest^28^.

### Characterisation of CH–π contacts

The CH–π contacts were further characterised by experimental and theoretical analyses. The COR molecule possesses 10 hydrogen atoms at the bowl periphery, and crystal data of [4]CC⊃(COR)_2_ and [4]CC⊃(COR-*d*_10_)_2_ respectively comprise three COR_cent_ orientations in two crystallographically independent bowl-in-tube structures (Supplementary Fig. 8). Thus, two sets of crystal data provided 120 data points of the CH–π contact in total and were suitable for detailed structural analyses with CH–π distances (*D*_pln_) and angles (*α*)^2^. As shown in the *D*_pln_-*α* plots (Fig. 3b), the CH–π contacts were scattered in the range of *D*_pln_ = 2.47-3.19 Å and *α* = 134–176°, respectively, which afforded average values of *D*_pln_ = 2.75 ± 0.16 Å and *α* = 157 ± 10°. Typical CH–π hydrogen bonds were reported to have average measures of *D*_pln_ = 2.73 ± 0.11 Å and *α* = 148 ± 11°, and, together with similar *D*_pln_-*α* scatter plots, indicated that the CH–π contacts in the bowl-in-tube complex were within the expected range of the CH–π hydrogen bond^2,29^. The *D*_pln_-*α* scatter plot of the DFT-optimised geometry of Me-[4]CC⊃COR also showed that the calculated CH–π contacts reproduced the experimental CH–π contacts of COR_cent_ molecules in the crystal structures (Fig. 3c). Thus, we believe that the COR_cent_ molecule with CH–π contacts also represents the solution phase structure of [4]CC⊃COR. Although the hydrogen bonds are intrinsically directional forces^1,2^, the unique tubular structure of the host should allow relayed relocations of the CH–π hydrogen bonds on the smoothly curved π-surface and, consequently, single-axis rotation (see below)^30^.

### Solid-state single-axis rotations of bowls in a tube

**Fig. 5:**
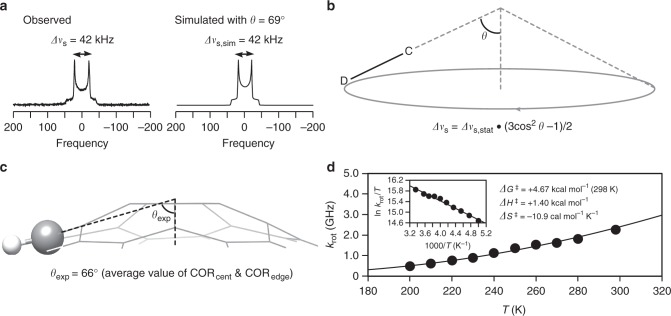
Solid-state single-axis rotations of the bowl guests in the tubular host. **a** Observed and simulated ^2^H NMR spectra of COR-*d*_10_ in [4]CC (298 K). **b** A cone model of single-axis rotations of a CD bond at a cone angle of *θ* for static quadrupole echo ^2^H NMR spectra. **c** A molecular structure of COR showing a representative experimental cone angle (*θ*_exp_) measured from a crystal structure (CCDC 1829017). The single-axis rotations around the tube axis were assumed for this cone angle. **d** Temperature-dependent rotational frequency of COR in [4]CC. Inset shows the Eyring plot for the energetic analyses

The single-axis rotation of the bowl guest in the tubular host was confirmed by solid-state NMR analysis (Fig. 5). A solid specimen of [4]CC⊃(COR-*d*_10_)_2_ was obtained from a 1:2 mixture of [4]CC and COR-*d*_10_ in CHCl_3_/2-propanol and was subjected to solid-state ^2^H NMR analysis (Methods section). The ^2^H NMR spectra were recorded under quadrupolar echo conditions with and without the use of magic angle spinning (MAS)^31^. As shown in Fig. 5 and Supplementary Fig. 11, a single resonance was commonly observed in the ^2^H NMR spectra, indicating the equivalency of COR molecules. The single Pake doublet further confirmed the presence of monotypic dynamics of COR molecules, as we should expect two overlapping doublets for a mixture of motionless and dynamic molecules^32^. In addition, although a large ^2^H quadrupolar splitting of 135 kHz (= *Δ****v***_s,stat_) was expected for deuterons on static motionless molecules^33,34^, the splitting of the observed Pake doublet was 42 kHz (= *Δ****v***_s_) (Fig. 5a). The observations indicated the presence of dynamic motions of COR molecules, and its biased non-isotropic motion was further clarified^35^.

A comparison of simulations with experimental data allowed us to disclose the single-axis rotations of COR molecules. The ^2^H quadrupolar splitting (*Δ****v***_s_) is under the influence of the molecular motions in the solid state^34^, and, for CD bonds that are rapidly rotating around a single axis in a cone model shown in Fig. 5b, the *Δ****v***_s_ value is given by the equation, *Δ****v***_s_ = *Δ****v***_s,stat_•(3cos^2^*θ* – 1)/2 (Supplementary Fig. 12; ref. ^33^). The cone angle-dependent splitting shows that the experimental splitting value of 42 kHz can thus be reproduced at the cone angles (*θ*) of 42° and 69°. Experimentally, under assumption of single-axis rotations of COR molecules (COR_cent_ and COR_edge_) around the tube axis of the [4]CC host in the crystal, the cone angles (*θ*_exp_) were measured from the crystal structures to afford an average angle of 66° (Fig. 5c). This experimental value, *θ*_exp_ = 66°, matched well with one of the simulation angles (*θ* = 69°). In addition, when we simulated the ^2^H spectrum with the cone angle (*θ* = 69°) by using NMR-WEBLAB, the spectrum was nicely reproduced (Fig. 5a; Methods section)^33^. Therefore, we conclude that the COR bowl guests in the [4]CC tubular host anisotropically rotate around the tube axis. Achieving single-axis rotations of polycyclic aromatic hydrocarbons with concyclic CH–π anchors demonstrates a unique way to control the directionality of solid-state molecular motions^5,26^.

Finally, the energetics of the single-axis rotation were disclosed. Kinetic analysis first failed with variable-temperature (VT) NMR measurements. The Pake doublet resonance from the ^2^H resonance was not affected by the temperature (Supplementary Fig. 13). Thus, even at the lowest temperature of our NMR instrument (200 K), the COR molecules were rotating rapidly without affecting the resonance shape^33^. We then adopted a kinetic analysis with spin–lattice relaxation time (*T*_1_) measurements to determine correlation time (*τ*_rot_) and rotational frequency (*k*_rot_ = *τ*_rot_^–1^)^36^. Decays in the saturation-recovery *T*_1_ measurements were successfully fitted by a single exponential curve with reliable goodness-of-fit coefficients (*R*^2^ > 0.99) to afford the correlation time (Supplementary Fig. 14; see also Methods section). In this measurement, the monotypic dynamics of COR molecules was also confirmed, as we did not observe a two-component decay^37^. At 298 K, for instance, the correlation time and the rotational frequency were 442 ps and 2.26 GHz, respectively, which confirmed the rapid single-axis rotation of the bowl guests^5,32^. The kinetic data were obtained for a temperature range between 200 to 298 K, and the Eyring plot in the form of 1/*T*-ln (*k*_rot_/*T*) allowed us to reveal the energy barriers of *ΔG*^‡^ = +4.67 kcal mol^–1^, *ΔH*^‡^ = +1.40 kcal mol^–1^ and –*TΔS*^‡^ = +3.27 kcal mol^–1^ (298 K) (Fig. 5d). The enthalpy barrier originates from reorientations of CH–π hydrogen bonds, which is minimised by a smooth uninterrupted surface of the tube for relayed reorientations^26^. The high entropy barrier in terms of –*TΔS*^‡^ for the bowl guests shows that consideration of the entropy cost is particularly important to design directional motions of molecules.

## Discussion

Microscopic observations have previously demonstrated the presence of supramolecular composites of organic molecules in single-wall carbon nanotubes (SWNT)^38–40^. Although the CH–π hydrogen bonds have not been considered for these composites, the present study with finite SWNT molecules suggests that the encapsulation could be driven by multiple, weak CH–π contacts in an enthalpically favourable manner. Interestingly, despite the 10 CH–π hydrogen bonds anchoring the bowl-shaped guest to the tubular host, the guest was given a freedom of rotation. Furthermore, the rotation was biased for single-axis motions with the aid of concyclic arrangements of CH–π contacts that enabled relayed reorientations of hydrogen bonds. This design principle embedded in this molecular system resembles to that adopted for ball bearings^41^. We believe that the CH–π arrays could be utilised as fundamental elements for molecular machinery in the future. Additionally, the introduction of axial anisotropy with guests in helical hosts is another interesting feature that needs to be fully exploited for chirality-induced functions such as chiroptical properties^42^. We are currently pursuing research exploring supramolecular systems with the CH–π assembly.

## Methods

### Materials

The [4]CC host was synthesised by a method reported in the literature^8^. The synthesis of the deuterated COR-*d*_10_ guest with a method reported in the literature was not fully successful (80% yield, >98%D)^15^, but after re-optimising the conditions, we obtained the compound in an acceptable yield (23% yield, 80%D) by performing the reaction at 130 °C.

### Job plot analysis

The solution phase stoichiometry of [4]CC and COR in the bowl-in-tube complex was determined by the Job plot analysis with ^1^H NMR spectra in dichloromethane-*d*_2_ at 298 K on a JEOL JNM-ECA II 600 equipped with the UltraCOOL probe. The total concentrations for the analyses were 1.09 mM. Representative spectra are shown in Fig. 2a and Supplementary Fig. 1, and the Job plots are shown in Fig. 2b.

### In-and-out exchange behaviours

The in-and-out equilibrium of [4]CC⊃COR was analysed by using VT ^1^H NMR spectra. A 1:2 mixture of [4]CC (0.333 mM) and COR (0.667 mM) in dichloromethane-*d*_2_ was prepared, and ^1^H NMR spectra were recorded in a temperature range of 298–179 K (Supplementary Fig. 2). At 298 K, a single resonance of COR was observed at 6.54 ppm, i.e., the weighted average chemical shift of the bound and unbound resonances^43^. When the temperature was lowered, the COR signal split, with a coalescence temperature of 243 K, into two resonances of bound (3.57 ppm at 179 K) and unbound (7.72 ppm at 179 K) species. The rate constant of the dissociation process, *k*_out_, is derived by

1 $$k_{\mathrm{out}} = \frac{\pi }{{\sqrt 2 }}\Delta \nu ,$$

where *Δv* is the resonance difference of bound and unbound COR at the lowest temperature^11,12^. The experimental value, *Δv* = 2.49 kHz (179 K), afforded *k*_out_ = 5.53 × 10^3^ s^–1^. Applying the *k*_out_ value in the Eyring equation

2 $$\Delta G^\ddagger = RT\ln \left( {\frac{{k_{\rm{B}}T}}{{hk_{{\rm{out}}}}}} \right),$$

where *R*, *T*, *k*_B_, *h* are the gas constant, the temperature, the Boltzmann constant and the Planck constant, respectively, we obtained *ΔG*^‡^ = –11.0 kcal mol^–1^.

### Isothermal titration calorimetry

The association constant (*K*_a_) of [4]CC⊃COR in dichloromethane was measured by the ITC analysis using a Malvern MicroCal iTC200 microcalorimeter. The ITC analysis also afforded *ΔG*, *ΔH* and *ΔS* for the complexation. Prior to the titration, the concentrations of [4]CC, COR and COR-*d*_10_ were calibrated by the UV-vis absorbance. To a solution of [4]CC (0.523 mM) in the microcalorimeter, a solution of COR (23.2 mM) or COR-*d*_10_ (22.1 mM) was added to derive the titration curves (Fig. 2c and Supplementary Fig. 4). The curves were then fitted by the ORIGIN software provided with the instrument. Titration experiments were performed five times for each specimen, and the averaged values with the standard deviation were reported.

### DFT calculations

Theoretical calculations were performed at the DFT level with the LC-BLYP functional^44^ and the 6-311 G(d) basis set^45^ on Gaussian 09^46^, and the results were compared with other functional (Supplementary Fig. 5). The association energy was derived by a procedure reported in the literature with the counterpoise BSSE corrections and the PCM solvation^17^. The analyses with the AIM method and electron density difference maps were performed with Multiwfn^47^.

### Crystallographic analysis

When we grew crystals from 1:1 mixtures of [4]CC and COR under various conditions, the crystalline-like particles were obtained in some instances. However, from these particles, we could not obtain diffraction data of sufficient quality. Single crystals of [4]CC⊃(COR)_2_ and [4]CC⊃(COR-*d*_10_)_2_ for X-ray crystallographic analysis were respectively obtained by slow evaporation of chloroform/2-propanol (~1:1 vol/vol) solution of [4]CC and the guest at 25 °C. The mixed ratios of [4]CC and COR were 1:10 and 1:2 for the crystals of [4]CC⊃(COR)_2_ and [4]CC⊃(COR-*d*_10_)_2_, respectively. Each single crystal was mounted on a thin polymer tip with cryoprotectant oil and frozen via flash cooling. The diffraction analysis of a single crystal with synchrotron X-ray sources was conducted at –178 °C by using a diffractometer equipped with a Rayonix MX225HE CCD detector at SPring-8 (BL38B1). The diffraction data were processed with the HKL2000 software programme^48^. The structure was solved by direct methods with the SHELXT software programme^49^ and refined by full-matrix least-squares on *F*^2^ using the SHELXL-2014/7 programme suite^50^ running with the Yadokari-XG 2009 software programme^51^. For the diffraction analyses, we examined two resolution cutoff conditions, <*I*/*σ*> = 1.3 (resolution = 0.93 Å) and a minimum cutoff at <*I*/*σ*> = 1.1 (resolution = 0.83 Å),^52^ to find qualitatively identical CH–π structures (Supplementary Fig. 9 and 10). Considering its superior confidence level (*R*_1_ = 9.68% vs. 8.86%), we adopted crystallographic data from <*I*/*σ*> = 1.1 after SQUEEZE treatments for the main discussion. In the structure refinement, certain restraints (SIMU, DFIX and DANG) were necessary to model reasonable structures for the system containing complex disordered structures. In short, SIMU restrains anisotropic displacement parameters by forcing similar values on neighbouring atoms, whereas DFIX and DANG restraints fix the bond lengths (C-C with DFIX and C-C-C with DANG). These restraints were applied to COR molecules and alkyl chains. Despite our extensive efforts to locate other disordered structures, we did not find superior models. For instance, inverted COR molecules were examined with the minute residual electron densities, but the current data failed to locate them with a sufficient confidence level. Non-hydrogen atoms were analysed anisotropically, and hydrogen and deuterium atoms were input at the calculated positions and refined with a riding model. The electron density attributed to solvent molecules was not modelled due to the severe disorders, and the structures were refined by using the PLATON/SQUEEZE protocol^53,54^. Under <*I*/*σ*> = 1.3, one level-A and two level-B alerts were suggested for [4]CC⊃(COR)_2_ by the PLATON/CIF check programme. The level-A alert indicated a lack of high-angle diffractions to result in a small sin*θ*_max_/wavelength value below 0.550 (0.5378), and the level-B alerts originated from slightly insufficient numbers of diffraction data. Under <*I*/*σ*> = 1.1, one level-A alert and three level-B alerts were suggested. The level-A alert indicated a low completeness (measured fraction by *θ*_full_ = 0.910) that is inherent to the low cutoff condition. The three level-B alerts were suggested for [4]CC⊃(COR-*d*_10_)_2_ by the PLATON/CIF check programme. One level-B alert was due to missed diffractions at the timing of beam stops and was an inherent problem of the present experimental environment. Two other level-B alerts originated from slightly insufficient numbers of diffraction data. These level-A and level-B alerts from insufficient numbers of diffractions are unavoidable for the highly complex structures with the severe disordered structures, because the disorders are present with the solvent molecules, alkyl group conformations, and corannulene orientations. In addition, under diffraction conditions (100 K), the rotation barrier for COR molecules is low (*ΔG*^‡^ = +2.49 kcal mol^–1^), which results in a rotational frequency of *k*_rot_ = 7.5 MHz. Nonetheless, the monochromated synchrotron source allowed us to refine the diffraction data with a resolution of 0.93/0.83 Å, which afforded reliable structures suitable for the present discussion and conclusions. The average standard uncertainty of C-C bonds, for instance, is 0.0130 Å for [4]CC⊃(COR)_2_ and 0.0101 Å for [4]CC⊃(COR-*d*_10_)_2_. Electron density mappings were generated on a COOT software programme^55^. The Hirshfeld surface analyses were performed using the CrystalExplorer software programme^25^, and the CH–π distances and angles were measured on Mercury CSD programme^56^. The crystal structures of [4]CC⊃(COR)_2_ and [4]CC⊃(COR-*d*_10_)_2_ are shown in Fig. 4 and Supplementary Fig. 8, respectively. Crystal data are summarised in Supplementary Tables 1-6.

### Solid-state ^2^H NMR analysis

A mixture of [4]CC (28 mg, 18 µmol) and COR-*d*_10_ (10 mg, 36 µmol) in CHCl_3_ (9 mL) and 2-propanol (45 mL) was stored in a partly open vial at 25 °C for 1 month with solvents being evaporated slowly. The method was essentially identical to the one adopted to obtain a single crystal of [4]CC⊃(COR-*d*_10_)_2_. Crystalline precipitates were collected by filtration and, without drying in vacuo, were transferred to a 3.2-mm ZrO_2_ NMR tube for the solid-state ^2^H NMR analysis. Solid-state ^2^H NMR spectroscopy was performed with a 3.2-mm HXMAS probe on JEOL JNM-ECA II 600. Spectra were recorded under static and MAS conditions (Fig. 5, Supplementary Fig. 11 and 13) with a quadrupolar echo sequence^31^. The static spectrum was simulated by the line shape simulation with NMR WEBLAB^33^ adopting parameters of *Δ****v***_s,sim_ = 135 kHz, *θ* = 69° and *η* = 0. The spin–lattice relaxation time *T*_1_ was measured by a saturation-recovery method in a temperature range of 200–298 K under static conditions without MAS^57^. The recovery data were fitted by a single exponential curve for the *T*_1_ fitting resulting in a high-quality coefficient of determination (*R*^2^ > 0.99; Supplementary Fig. 14). The successful curve fitting with the single exponential curve showed that despite the presence of multiple, crystallographically inequivalent COR molecules, their dynamic motions spectroscopically appeared as monotypic motions. Representative examples of *T*_1_ data are shown in Supplementary Table 7. The rotational correlation time *τ*_rot_ was calculated from the literature-reported equation for a single-axis rotation model (Eq. (14) in ref. ^31^) by using the experimental *T*_1_ value in addition to *Δν*_s,sim_ = 135 kHz, *θ* = 69°, *η* = 0 and *ω* = 92.1 MHz.

## Electronic supplementary material

Supplementary Information

Description of Additional Supplementary Files

Supplementary Data 1

Supplementary Data 2

## Data Availability

Crystallographic data are available at Cambridge Crystallographic Database Centre (https://www.ccdc.cam.ac.uk) as CCDC1829014, 1829015 (<*I*/*σ*> = 1.3), 1829016, 1829017 (COR-*d*_10_), 1854917 and 1854918 (<*I*/*σ*> = 1.1). All other data that support the findings of this study are available from the corresponding author upon reasonable request.
