# Author Correction: Concyclic CH-π arrays for single-axis rotations of a bowl in a tube

**DOI:** 10.1038/s41467-020-15286-w

**Published:** 2020-03-31

**Authors:** Taisuke Matsuno, Masahiro Fujita, Kengo Fukunaga, Sota Sato, Hiroyuki Isobe

**Affiliations:** 1 Department of Chemistry, The University of Tokyo, Hongo 7-3-1, Bunkyo-ku, Tokyo, 113-0033 Japan; 2 JST, ERATO, Isobe Degenerate π-Integration Project, Hongo 7-3-1, Bunkyo-ku, Tokyo, 113-0033 Japan; 3 Department of Chemistry, Tohoku University, Aoba-ku, Sendai, 980-8578 Japan

**Keywords:** Supramolecular chemistry, Carbon nanotubes and fullerenes, Molecular machines and motors

Correction to: *Nature Communications* 10.1038/s41467-018-06270-6, published online 17 September 2018

The original version of this Article contained errors in values of Δ_rot_, *k*_rot_, Δ*G*^‡^, Δ*H*^‡^, Δ*S*^‡^ and –*T*Δ*S*^‡^ in the last paragraph of the Results section. An error in the eighth sentence of the last paragraph of the Results incorrectly read ‘442 ps and 2.26 GHz’. The correct version states ‘11.1 ps and 89.9 GHz’. An error in the ninth sentence of the last paragraph incorrectly read ‘Δ*G*^‡^ = +4.67  kcal mol^–1^, Δ*H*^‡^ = +1.40 kcal mol^–1^ and −*T*Δ*S*^‡^ = +3.27 kcal mol^–1^’. The correct version states ‘Δ*G*^‡^ = +2.50 kcal mol^–1^, Δ*H*^‡^ = +1.28 kcal mol^–1^ and −*T*Δ*S*^‡^ = +1.22 kcal mol^–1^’. This has been corrected in the PDF and HTML versions of the Article.

The original version of this Article also contained an error in Fig. 5d, in which incorrect values were given for Δ*G*^‡^, Δ*H*^‡^, Δ*S*^‡^, and the scales of the *y*-axes in both the main graph and the inset graph were incorrect. The correct version of Fig. 5d is:

which replaces the previous incorrect version:



This has been corrected in the PDF and HTML versions of the Article.

The original version of the Supplementary Information associated with this Article contained an error in Supplementary Table 7, in which incorrect values were given in the third and fourth columns from the left. The correct version of Supplementary Table 7 is:

**Table 1:** 

Temperature	*T*_1_ (sec)	*τ*_rot_ (psec)	1/*τ*_rot_=*k*_rot_ (GHz)
298 K	0.207	11.1	89.9
280 K	0.166	13.9	71.9
270 K	0.148	15.6	64.2
260 K	0.141	16.3	61.4
250 K	0.125	18.4	54.4
240 K	0.104	22.1	45.3
230 K	0.0845	27.2	36.7
220 K	0.0718	32.1	31.2
210 K	0.0592	38.9	25.7
200 K	0.0484	47.5	21.0

which replaces the previous incorrect version:

**Table 2:** 

Temperature	*T*_1_ (sec)	*τ* (nsec)	1/*τ* =*k*_rot_ (GHz)
298 K	0.207	0.442	2.26
280 K	0.166	0.554	1.81
270 K	0.148	0.621	1.61
260 K	0.141	0.651	1.54
250 K	0.125	0.736	1.36
240 K	0.104	0.892	1.12
230 K	0.0845	1.11	0.898
220 K	0.0718	1.33	0.752
210 K	0.0592	1.65	0.605
200 K	0.0484	2.11	0.473

The HTML has been updated to include a corrected version of the Supplementary Information.

