# Supplementary material for: Concyclic CH-π arrays for single-axis rotations of a bowl in a tube
Source: Nat Commun. 2018 Sep 17;9:3779. doi: 10.1038/s41467-018-06270-6 (PMC6141547; doi:10.1038/s41467-018-06270-6)
Supplement: Supplementary file 1 — Supplementary Information [file 41467_2018_6270_MOESM1_ESM.pdf]

Supplementary Information

Concyclic CH-π arrays for single-axis rotations of a bowl in a tube

Taisuke Matsuno, Masahiro Fujita, Kengo Fukunaga, Sota Sato, Hiroyuki Isobe*

*e-mail: isobe@chem.s.u-tokyo.ac.jp

Table of Contents

Supplementary Figures 2

Job plot analysis by ^{1}H NMR spectroscopy 2

ITC analysis of [4]CC⊃COR-d_{10} 4

Theoretical calculations 5

Solid-state structures of 1:2 complexes 7

Solid-state ^{2}H NMR analyses 10

Supplementary Tables 13

Crystallographic analyses 13

Solid-state ^{2}H NMR analyses 19

Supplementary Figures

Job plot analysis by ¹H NMR spectroscopy

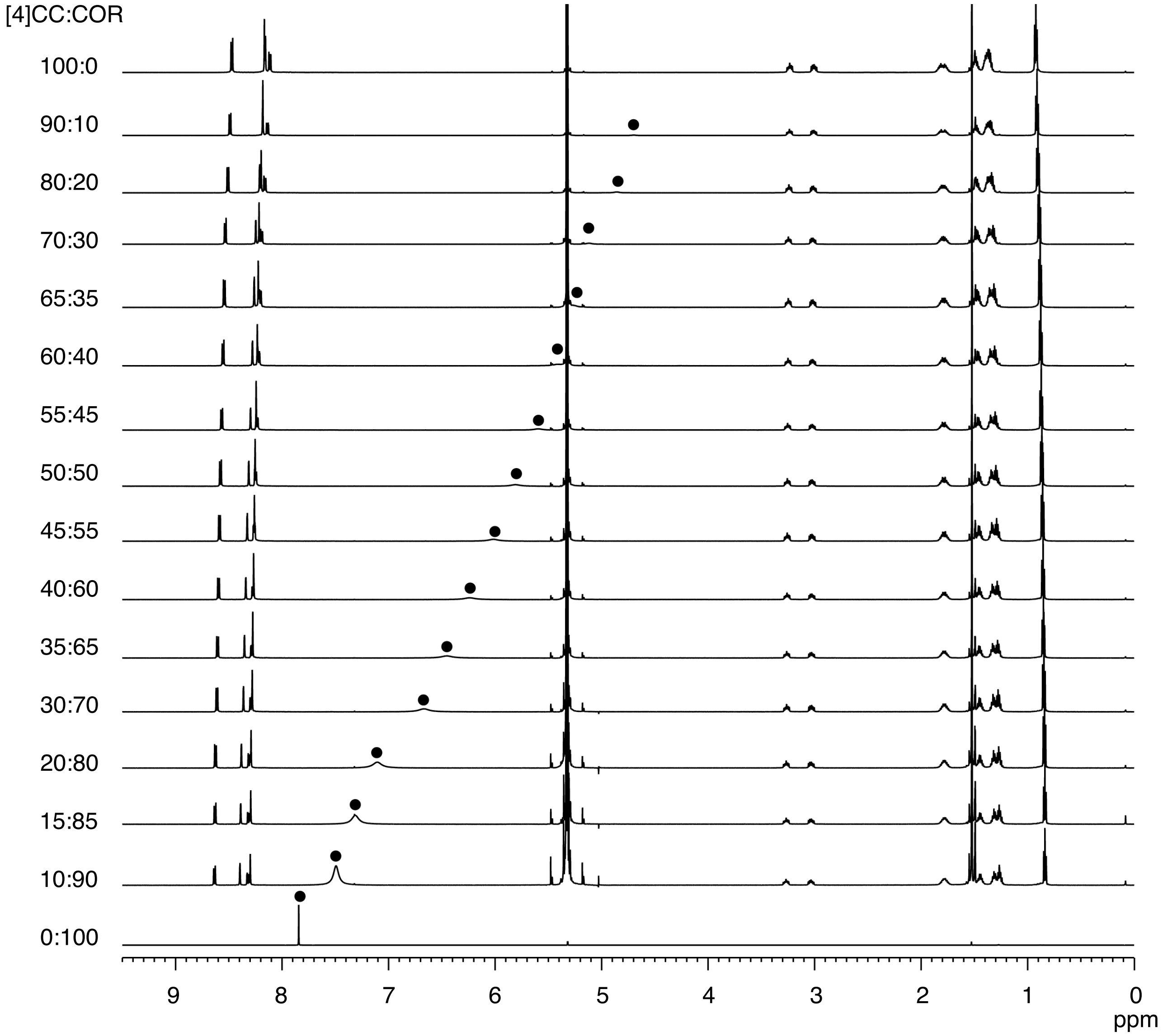

Supplementary Figure 1. ^{1}H NMR spectra of [4]CC⊃COR in CD_2Cl_2 at 298 K for the Job plot analysis. Total concentration = 1.09 mM. Resonances of COR are labelled with dots.

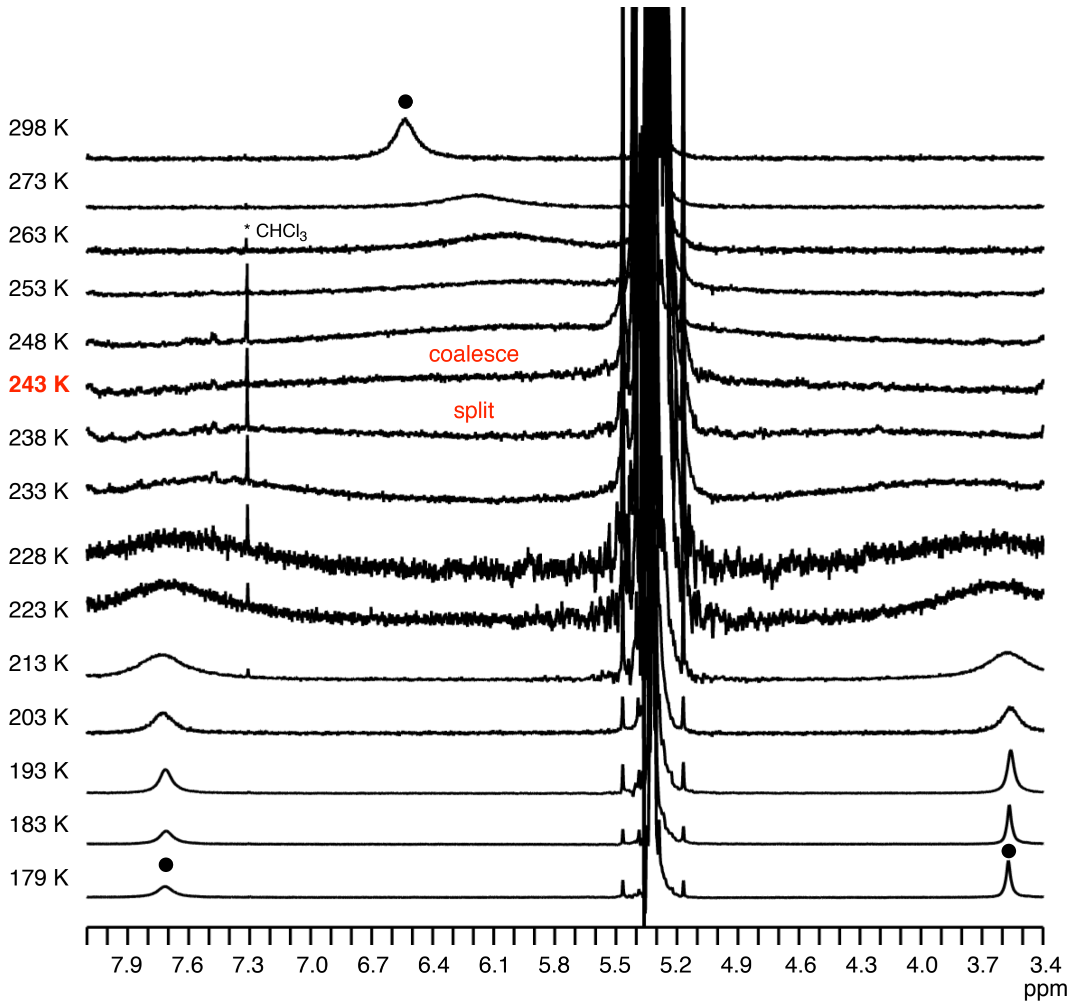

Supplementary Figure 2. VT ^{1}H NMR spectra of 1:2 mixture of [4]CC and COR in CD_2Cl_2. Resonances of COR at the highest and lowest temperatures are labelled with dots. A resonance of residual chloroform became observable, when the resonance intensity of the specimen got lowered due to slow-exchange broadening.

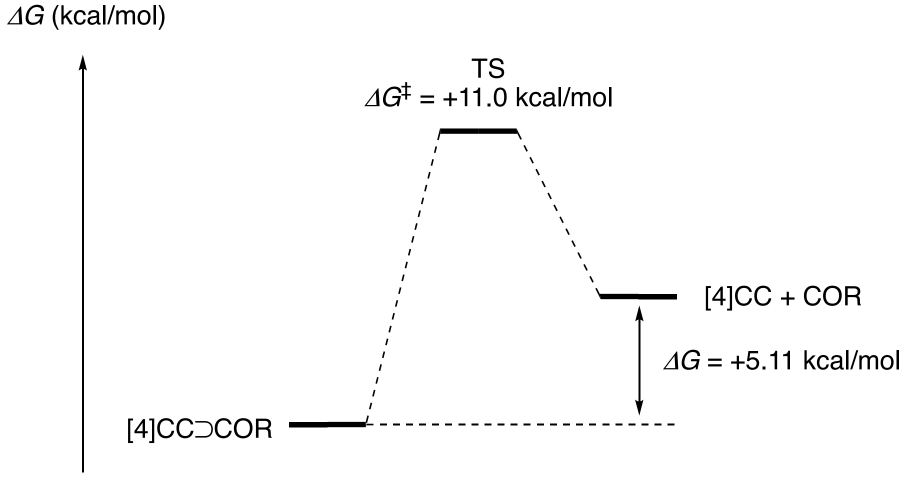

Supplementary Figure 3. An energy profile of the in-and-out process of [4]CC⊃COR in dichloromethane at 243 K. The energy difference (ΔG) between bound and unbound states is estimated by the ITC analysis.

ITC analysis of [4]CC⊃COR-d_{10}

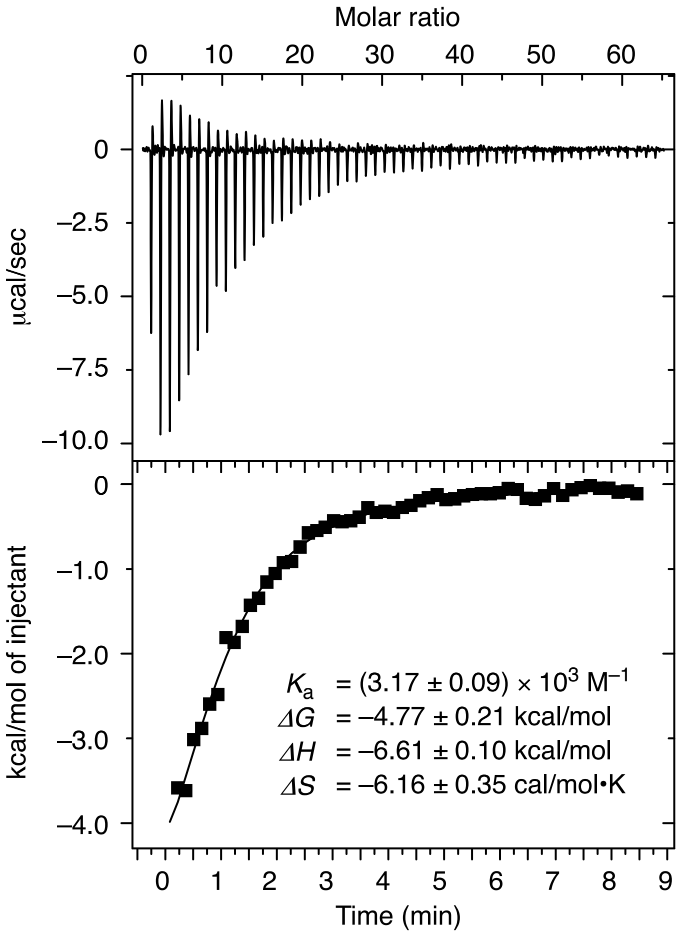

Supplementary Figure 4. Representative ITC data for [4]CC⊃COR-d_{10}.

Theoretical calculations

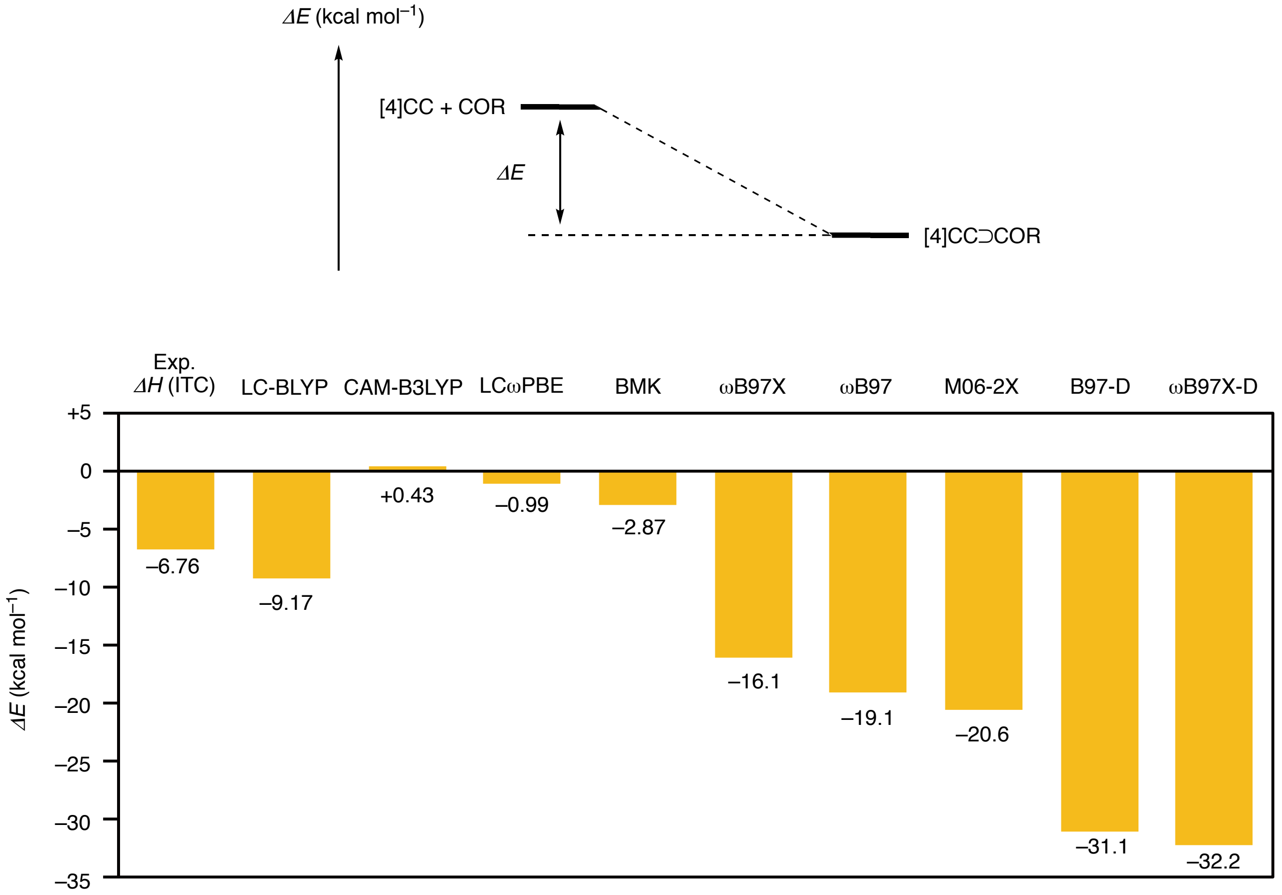

Supplementary Figure 5. DFT calculations of the association energy of Me-[4]CC⊃COR with the basis set of 6-311G(d) after the BSSE/PCM treatment.

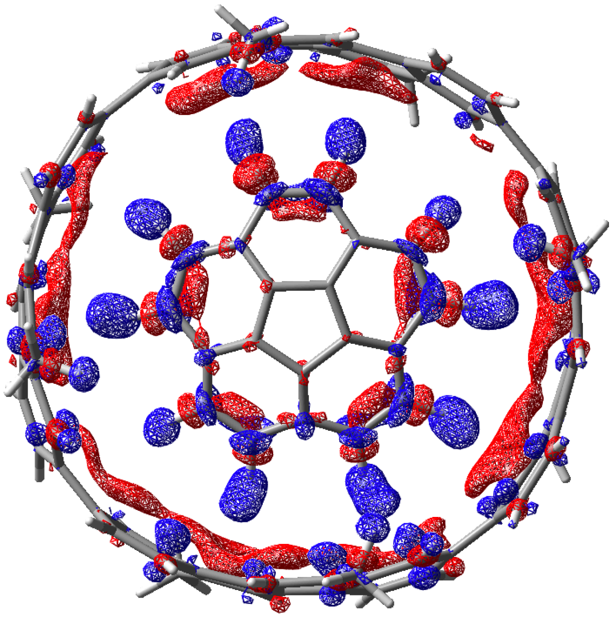

Supplementary Figure 6. The electron density difference maps of [4]CC⊃COR [LC-BLYP/6-311G(d), isosurface = 0.0003 e]. Residues in blue indicate the decrease of electron density, whereas residues in red indicate the increase of electron density.

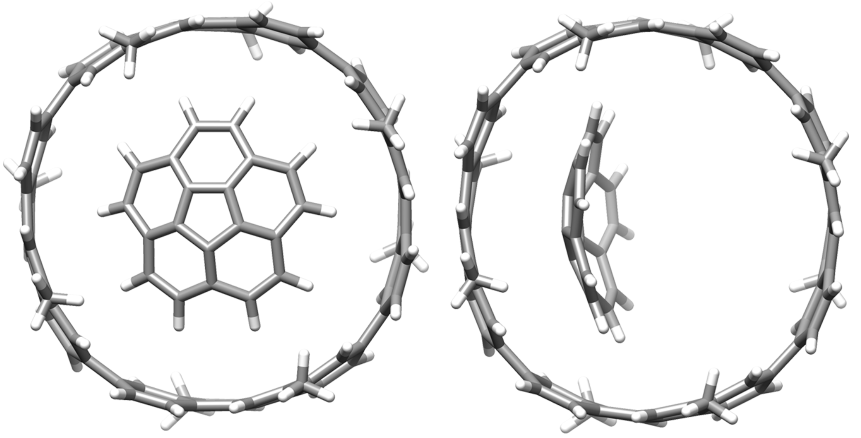

bowl-in-tube structure
δ_{ave} = 3.86 ppm
(2.89-4.94 ppm)
ΔE = –9.17 kcal mol^{-1}

convex-concave structure
δ_{ave} = 5.99 ppm
(3.30-7.38 ppm)
ΔE = –4.08 kcal mol^{-1}

Supplementary Figure 7. DFT-GIAO calculations of the ^{1}H resonance in Me-[4]CC⊃COR. The geometries were optimised at the LC-BLYP/6-311G(d) level. The average COR resonance of the bowl-in-tube structure predicted 3.86 ppm, and that of the convex-concave structure predicted 5.99 ppm. The bowl-in-tube structure reproduced the experimental value of 3.57 ppm and was proven appropriate as the time-average structure. The *ΔE* values show the association energies after the BSSE/PCM treatment.

Solid-state structures of 1:2 complexes

a

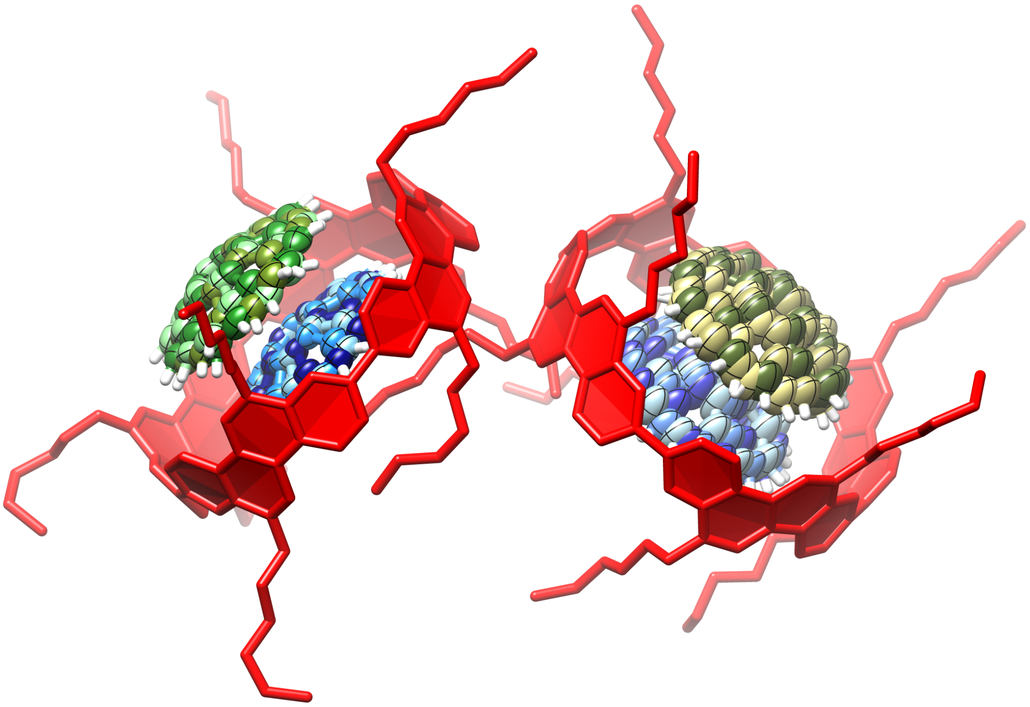

b

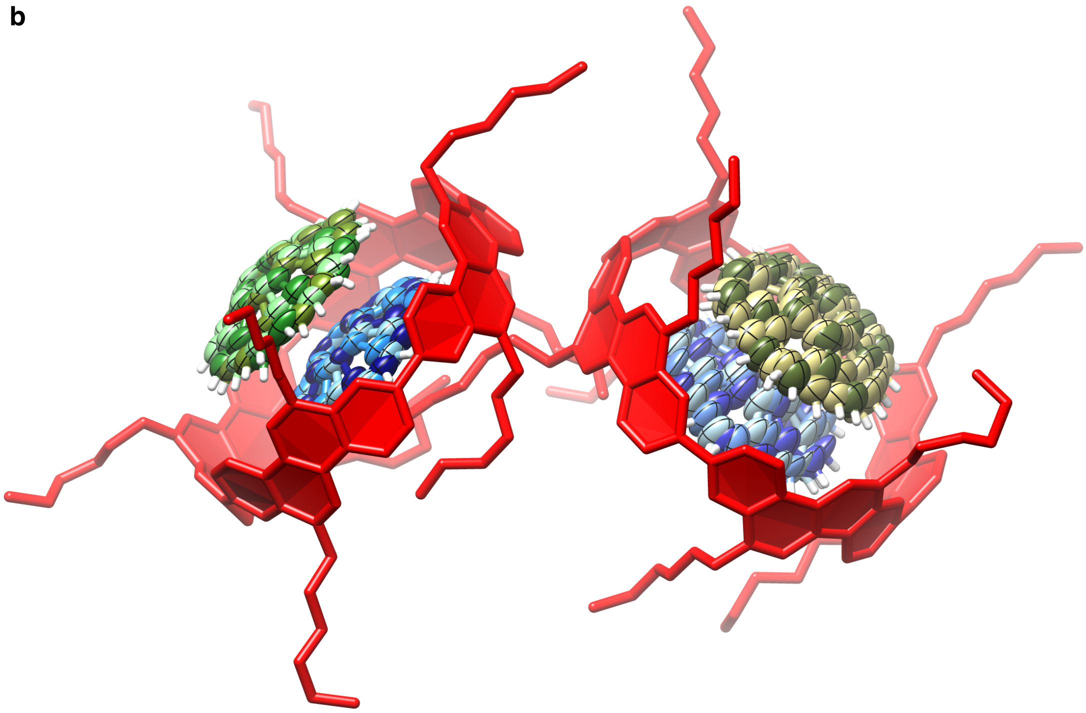

Supplementary Figure 8. Crystal structures of two crystallographically inequivalent complexes. Thermal ellipsoids of COR are scaled to enclose 50% probability, and one major structrues of alkyl conformers is shown. **a,** [4]CC$\supset$(COR)$_2$. **b,** [4]CC$\supset$(COR-d_{10})$_2$.

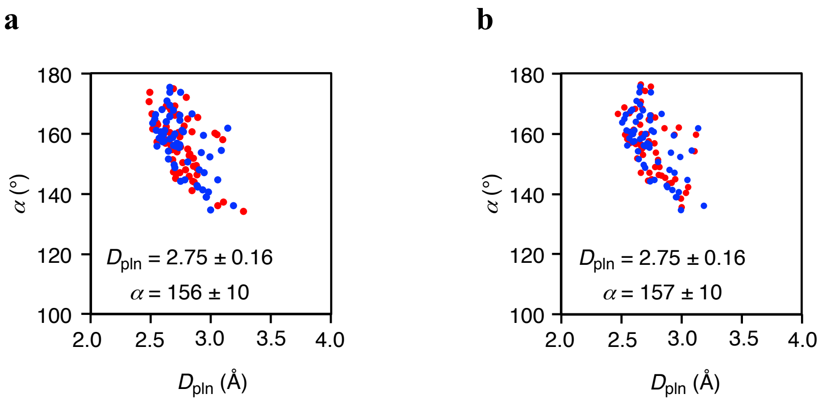

Supplementary Figure 9. Comparison of the resolution cutoff for the present analyses of CH-π contacts. **a,** <*I*/*σ*> = 1.3. **b,** <*I*/*σ*> = 1.1 (Same as Fig. 3b).

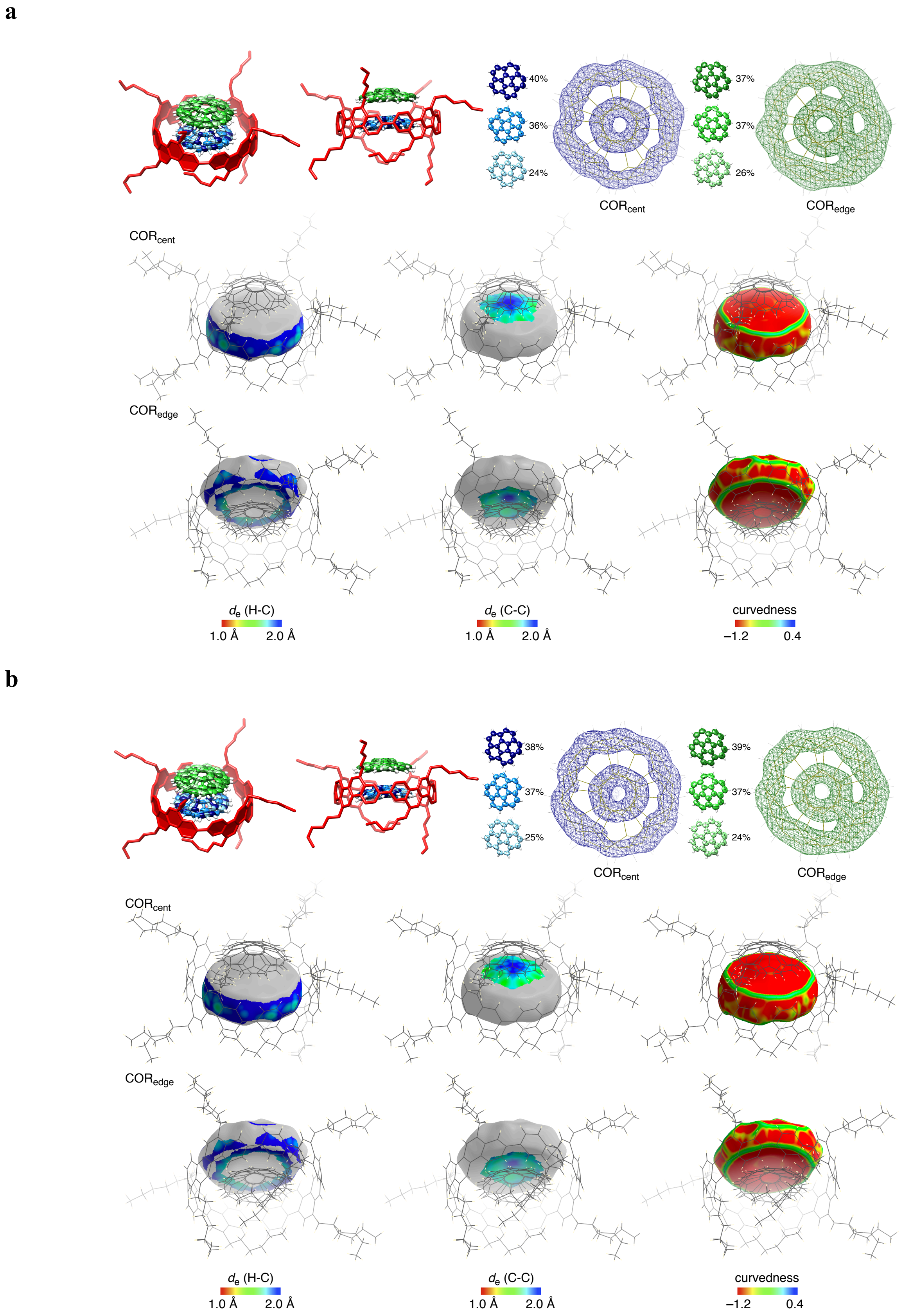

Supplementary Figure 10. Comparison of the resolution cutoff for the present analyses of molecular structures. **a,** $\langle I/\sigma \rangle$ = 1.3. **b,** $\langle I/\sigma \rangle$ = 1.1 (Same as Fig. 4).

Solid-state ^{2}H NMR analyses

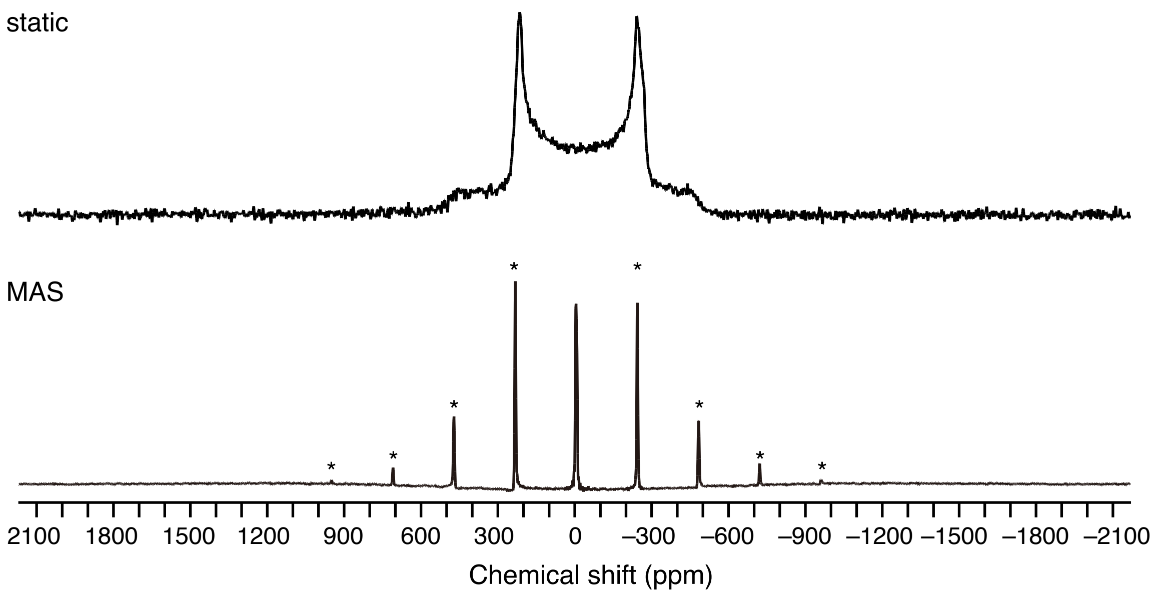

Supplementary Figure 11. Solid-state VT ^{2}H NMR spectra of [4]CC⊃(COR-d_{10})$_2$ under MAS conditions (22 kHz; 298 K). Asterisks in the MAS spectrum denote spinning sidebands that appear at the spinning frequency of 22 kHz. The static spectrum is shown as a reference and is identical to those appear in Fig. 5 and Supplementary Fig. 13.

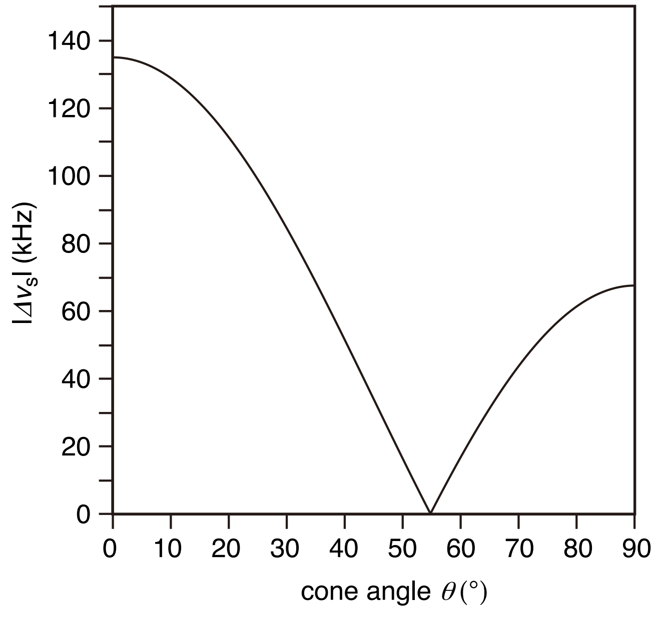

Supplementary Figure 12. Angle-dependent quadrupolar splitting ($|\Delta\nu_s|$) in static solid-state ^{2}H NMR spectra for deuteron under single-axis rotations. The graph is given by an equation, $\Delta\nu_s = \Delta\nu_{s,stat} \bullet (3\cos^2\theta - 1)/2$, with the $\Delta\nu_{s,stat}$ value of 135 kHz.

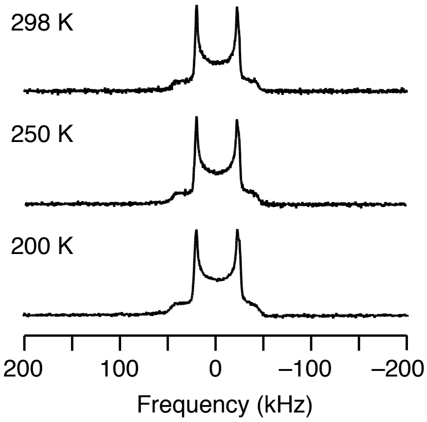

Supplementary Figure 13. Solid-state VT ^2H NMR spectra of [4]CC⊃(COR-d_{10})$_2$ under static conditions without MAS.

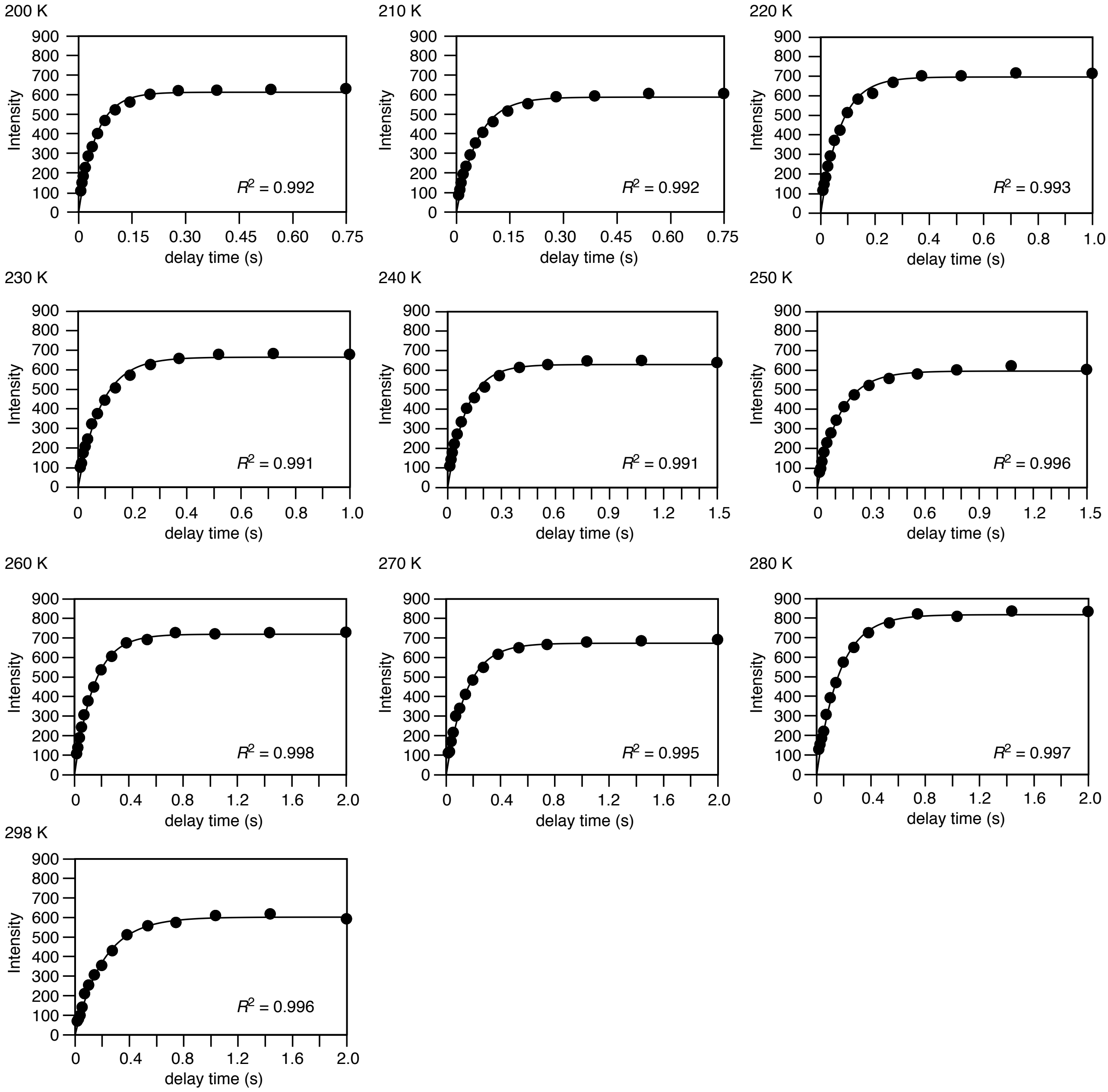

Supplementary Figure 14. Curve-fitting of ^{2}H saturation-recovery data. Intensity of the first points in FID were plotted and fitted with single exponential curves to afford T_1 values. The coefficients of determination in R^2 are shown for each single-exponential fitting.

Supplementary Tables

Crystallographic analyses

Supplementary Table 1. Crystal data and structure refinement for [4]CC⊃(COR)$_2$ without SQUEEZE. <*I*/*σ*> = 1.1.

CCDC No.	1854918
Empirical formula	C_{160} H_{156} $O_{7.50}$
Formula weight	2198.84
Temperature	100(2) K
Wavelength	0.850 Å
Crystal system	Trigonal
Space group	$P3_221$
Unit cell dimensions	a = 28.529(4) Å α = 90°.
	b = 28.529(4) Å β = 90°.
	c = 63.941(13) Å γ = 120°.
Volume	45070(16) Å^3
Z	12
Density (calculated)	0.972 Mg/m^3
Absorption coefficient	0.085 mm^{-1}
F(000)	14112
Crystal size	0.100 × 0.100 × 0.100 mm^3
Theta range for data collection	1.246 to 30.806°.
Index ranges	–32<=*h*<=31, –30<=*k*<=31, –77<=*l*<=77
Reflections collected	484437
Independent reflections	48612 [*R*(int) = 0.0572]
Completeness to theta = 30.664°	91.0 %
Absorption correction	Semi-empirical from equivalents
Max. and min. transmission	1.000 and 0.700
Refinement method	Full-matrix least-squares on F^2
Data / restraints / parameters	48612 / 9140 / 4976
Goodness-of-fit on F^2	1.241
Final *R* indices [*I*>2sigma(*I*)]	R_1 = 0.1120, wR_2 = 0.2783
R indices (all data)	R_1 = 0.1401, wR_2 = 0.3150
Absolute structure parameter	–2.6(3)
Extinction coefficient	n/a
Largest diff. peak and hole	0.439 and –0.347 e.Å^{-3}

Supplementary Table 2. Crystal data and structure refinement for [4]CC⊃(COR)$_2$ with SQUEEZE. $\langle I/\sigma \rangle$ = 1.1.

CCDC No.	1854917	
Empirical formula	$C_{160}H_{156}$	
Formula weight	2078.84	
Temperature	100(2) K	
Wavelength	0.850 Å	
Crystal system	Trigonal	
Space group	$P3_221$	
Unit cell dimensions	a = 28.529(4) Å	α = 90°.
	b = 28.529(4) Å	β = 90°.
	c = 63.941(13) Å	γ = 120°.
Volume	45070(16) Å^3	
Z	12	
Density (calculated)	0.919 Mg/m^3	
Absorption coefficient	0.074 mm^{-1}	
F(000)	13392	
Crystal size	0.100 × 0.100 × 0.100 mm^3	
Theta range for data collection	1.246 to 30.806°.	
Index ranges	$-32<=h<=31$, $-30<=k<=31$, $-77<=l<=77$	
Reflections collected	484466	
Independent reflections	48613 [R(int) = 0.0572]	
Completeness to theta = 30.664°	91.0 %	
Absorption correction	Semi-empirical from equivalents	
Max. and min. transmission	1.000 and 0.700	
Refinement method	Full-matrix least-squares on F^2	
Data / restraints / parameters	48613 / 9193 / 4855	
Goodness-of-fit on F^2	1.083	
Final R indices [I>2sigma(I)]	R_1 = 0.0886, wR_2 = 0.2390	
R indices (all data)	R_1 = 0.1143, wR_2 = 0.2774	
Absolute structure parameter	2.8(3)	
Extinction coefficient	n/a	
Largest diff. peak and hole	0.318 and –0.245 e.Å^{-3}	

Supplementary Table 3. Crystal data and structure refinement for [4]CC⊃(COR)$_2$ without SQUEEZE. <I/σ> = 1.3.

CCDC No.	1829014
Empirical formula	C_{320} H_{312} O_{15}
Formula weight	4397.68
Temperature	100(2) K
Wavelength	0.85000 Å
Crystal system	Trigonal
Space group	$P3_221$
Unit cell dimensions	a = 28.529(4) Å α = 90°.
	b = 28.529(4) Å β = 90°.
	c = 63.941(13) Å γ = 120°.
Volume	45070(16) Å^3
Z	6
Density (calculated)	0.972 Mg/m^3
Absorption coefficient	0.085 mm^{-1}
F(000)	14112
Crystal size	0.100 × 0.100 × 0.100 mm^3
Theta range for data collection	1.143 to 27.204°.
Index ranges	–30<=h<=30, –30<=k<=30, –68<=l<=68
Reflections collected	412865
Independent reflections	38839 [R(int) = 0.0523]
Completeness to theta = 27.204°	99.2 %
Absorption correction	Semi-empirical from equivalents
Max. and min. transmission	1.000 and 0.700
Refinement method	Full-matrix least-squares on F^2
Data / restraints / parameters	38839 / 9063 / 4874
Goodness-of-fit on F^2	1.292
Final R indices [I>2sigma(I)]	R_1 = 0.1145, wR_2 = 0.2782
R indices (all data)	R_1 = 0.1351, wR_2 = 0.3047
Absolute structure parameter	–2.6(3)
Extinction coefficient	n/a
Largest diff. peak and hole	0.482 and –0.442 e.Å^{-3}

Supplementary Table 4. Crystal data and structure refinement for [4]CC⊃(COR)$_2$ with SQUEEZE. <I/σ> = 1.3.

CCDC No.	1829015	
Empirical formula	C_{160} H_{156}	
Formula weight	2078.84	
Temperature	100(2) K	
Wavelength	0.85000 Å	
Crystal system	Trigonal	
Space group	$P3_221$	
Unit cell dimensions	a = 28.529(4) Å	α = 90°.
	b = 28.529(4) Å	β = 90°.
	c = 63.941(13) Å	γ = 120°.
Volume	45070(16) Å^3	
Z	12	
Density (calculated)	0.919 Mg/m^3	
Absorption coefficient	0.074 mm^{-1}	
F(000)	13392	
Crystal size	0.100 × 0.100 × 0.100 mm^3	
Theta range for data collection	1.143 to 27.204°.	
Index ranges	–30<=h<=30, –30<=k<=30, –68<=l<=68	
Reflections collected	412943	
Independent reflections	38846 [R(int) = 0.0522]	
Completeness to theta = 27.204°	99.2 %	
Absorption correction	Semi-empirical from equivalents	
Max. and min. transmission	1.000 and 0.700	
Refinement method	Full-matrix least-squares on F^2	
Data / restraints / parameters	38846 / 9056 / 4756	
Goodness-of-fit on F^2	1.189	
Final R indices [I>2sigma(I)]	R_1 = 0.0968, wR_2 = 0.2531	
R indices (all data)	R_1 = 0.1149, wR_2 = 0.2814	
Absolute structure parameter	2.4(3)	
Extinction coefficient	n/a	
Largest diff. peak and hole	0.345 and -0.275 e.Å^{-3}	

Supplementary Table 5. Crystal data and structure refinement for [4]CC⊃(COR-d_{10})$_2$ without SQUEEZE.

CCDC No.	1829016
Empirical formula	$C_{160} H_{139.60} D_{16.40} O_7$
Formula weight	2207.34
Temperature	100(2) K
Wavelength	0.90000 Å
Crystal system	Trigonal
Space group	$P3_221$
Unit cell dimensions	a = 28.739(4) Å α = 90°.
	b = 28.739(4) Å β = 90°.
	c = 63.925(13) Å γ = 120°.
Volume	45724(16) Å^3
Z	12
Density (calculated)	0.962 Mg/m^3
Absorption coefficient	0.095 mm^{-1}
F(000)	14064
Crystal size	0.100 × 0.100 × 0.100 mm^3
Theta range for data collection	1.210 to 31.971°.
Index ranges	–33<=h<=33, –33<=k<=33, –75<=l<=75
Reflections collected	516959
Independent reflections	51262 [R(int) = 0.0590]
Completeness to theta = 31.971°	98.7 %
Absorption correction	Semi-empirical from equivalents
Max. and min. transmission	1.000 and 0.501
Refinement method	Full-matrix least-squares on F^2
Data / restraints / parameters	51262 / 9184 / 4904
Goodness-of-fit on F^2	1.441
Final R indices [I>2sigma(I)]	R_1 = 0.1250, wR_2 = 0.3019
R indices (all data)	R_1 = 0.1429, wR_2 = 0.3370
Absolute structure parameter	–0.5(3)
Extinction coefficient	n/a
Largest diff. peak and hole	0.576 and –0.395 e.Å^{-3}

Supplementary Table 6. Crystal data and structure refinement for [4]CC⊃(COR-d_{10})$_2$ with SQUEEZE.

CCDC No.	1829017
Empirical formula	$C_{160} H_{139.60} D_{16.40}$
Formula weight	2095.34
Temperature	100(2) K
Wavelength	0.90000 Å
Crystal system	Trigonal
Space group	$P3_221$
Unit cell dimensions	a = 28.739(4) Å α = 90°.
	b = 28.739(4) Å β = 90°.
	c = 63.925(13) Å γ = 120°.
Volume	45724(16) $Å^3$
Z	12
Density (calculated)	0.913 Mg/m^3
Absorption coefficient	0.084 mm^{-1}
F(000)	13392
Crystal size	0.100 × 0.100 × 0.100 mm^3
Theta range for data collection	1.210 to 31.971°.
Index ranges	$-33<=h<=33$, $-33<=k<=33$, $-75<=l<=75$
Reflections collected	517011
Independent reflections	51269 [R(int) = 0.0590]
Completeness to theta = 31.971°	98.8 %
Absorption correction	Semi-empirical from equivalents
Max. and min. transmission	1.000 and 0.501
Refinement method	Full-matrix least-squares on F^2
Data / restraints / parameters	51269 / 9117 / 4792
Goodness-of-fit on F^2	1.318
Final R indices [I>2sigma(I)]	R_1 = 0.1012, wR_2 = 0.2706
R indices (all data)	R_1 = 0.1199, wR_2 = 0.3102
Absolute structure parameter	0.9(3)
Extinction coefficient	n/a
Largest diff. peak and hole	0.451 and –0.277 e.Å^{-3}

Solid-state ^{2}H NMR analyses

Supplementary Table 7. Rotational correlation time τ and rotational frequency k_{rot}.

Temperature	T_1 (sec)	τ (nsec)	$1/\tau = k_{rot}$ (GHz)
298 K	0.207	0.442	2.26
280 K	0.166	0.554	1.81
270 K	0.148	0.621	1.61
260 K	0.141	0.651	1.54
250 K	0.125	0.736	1.36
240 K	0.104	0.892	1.12
230 K	0.0845	1.11	0.898
220 K	0.0718	1.33	0.752
210 K	0.0592	1.65	0.605
200 K	0.0484	2.11	0.473
