# Supplementary material for: Concyclic CH-π arrays for single-axis rotations of a bowl in a tube
Source: Nat Commun. 2018 Sep 17;9:3779. doi: 10.1038/s41467-018-06270-6 (PMC6141547; doi:10.1038/s41467-018-06270-6)
Supplement: Supplementary file 2 — Description of Additional Supplementary Files [file 41467_2018_6270_MOESM2_ESM.pdf]

Description of Additional Supplementary Files

File Name: Supplementary Data 1
Description: Cartesian coordinates of the bowl-in-tube structure.

File Name: Supplementary Data 2
Description: Cartesian coordinates of the convex-concave structure.
